# Association between serum IgG concentrations and the incidence of infections in patients with chronic lymphocytic leukemia and secondary immunodeficiency under treatment with Privigen 

**DOI:** 10.5414/CP204473

**Published:** 2024-04-05

**Authors:** Burkhard Otremba, Ferdinand Haslbauer, Marcel Reiser, Rudolf Weide, Michael Obermeier, Dietmar Pfründer

**Affiliations:** 1 Center for Oncology, Oldenburg, Germany,; 2 Department of Internal Medicine, Salzkammergut Hospital, Vöcklabruck, Austria,; 3 Center for Oncology and Hematology, Cologne,; 4 Hematology/Oncology Group Practice, Koblenz,; 5 GKM Clinical Research Services, Munich, and; 6 CSL Behring, Hattersheim, Germany

**Keywords:** Privigen, intravenous immunoglobulin (IVIG), serum IgG concentrations, infections, chronic lymphocytic leukemia (CLL)

## Abstract

Objective: To investigate the association between serum immunoglobulin G (IgG) concentrations and the incidence of infections in patients with chronic lymphocytic leukemia (CLL) and secondary immunodeficiency receiving treatment with Privigen. Materials and methods: Data was analyzed from a non-interventional study conducted in 31 centers in Germany and 1 in Austria. Adult CLL patients with hypogammaglobulinemia and recurrent infections were allowed to enter the study upon signing informed consent, if a prior decision for treatment with Privigen had been made. All infections requiring an antimicrobial treatment were subject to analysis. Patients were stratified according to their mean post-baseline serum IgG trough levels in a group with lower IgG trough levels (≤ 5.0 g/L), and a group with higher IgG trough levels (> 5.0 g/L). Results: Overall, 89 patients and 840 treatment cycles were analyzed. Up to 11 treatment cycles (average duration 29 days) were documented in each patient. In the group with higher IgG trough levels (> 5.0 g/L, N = 72), significantly fewer infections were observed than in the group with lower IgG trough levels (≤ 5.0 g/L, N = 17), including fewer severe and serious infections. The Privigen dosage was a major determinant of the post-baseline serum IgG levels. Overall tolerability of Privigen was assessed as very good or good in 91% of patients. Conclusion: This analysis confirms the association of serum IgG trough levels with the incidence of infections and highlights the importance of careful monitoring of IgG levels during treatment of secondary immunodeficiencies in CLL patients.

**What is known about this subject**

- Patients with chronic lymphocytic leukemia (CLL) develop secondary immunodeficiencies due to changes in B-cell immunity including hypogammaglobulinemia.
- Several randomized trials have shown that substitution treatment with polyvalent intravenous immunoglobulins (IVIG) can reduce overall infections and particularly serious infections in CLL patients with hypogammaglobulinemia.

**What this study adds**

- This study provides data on the association between serum IgG concentrations and the incidence of infections in CLL patients receiving substitution treatment with IVIG.
- This study provides data on the effectiveness and tolerability of the IVIG Privigen (IgPro10; CSL Behring, King of Prussia, PA, USA) in the treatment of CLL patients with secondary immunodeficiency.

## Introduction 

Chronic lymphocytic leukemia (CLL) is the most common type of leukemia within the Western hemisphere, with an incidence of 6 – 7 cases per 100,000/year. CLL mainly affects people of advanced age; the median age at initial diagnosis is between 70 and 75 years old [1]. 

The disease is characterized by bone marrow infiltration and peripheral lymphocytosis, and leads to generalized lymphadenopathy, splenomegaly and hepatomegaly, worsening bone marrow insufficiency, and cytopenia in the further course of the disease. In the majority of cases, as the disease progresses, hypogammaglobulinemia also occurs, attributed to the functional impairment of T cells and non-clonal, CD5-negative B cells [2, 3]. Hypogammaglobulinemia is associated with a markedly increased risk of infections in CLL patients, including pneumonia, sinusitis, septicemia, and urinary tract infections [3]. These infections cause or contribute to 25 – 50% of deaths, and thus remain one of the most decisive factors for morbidity and mortality in CLL patients [2, 4, 5, 6]. 

As decreased immunoglobulin G (IgG) levels in CLL are associated with a higher risk of infections as well as a shorter survival, replacement treatment with intravenous immunoglobulin (IVIG) appears to be a logical treatment strategy [7, 8, 9]. In fact, the efficacy of IVIG in lowering infection rates, particularly of serious infections, has been shown in several randomized trials [10, 11, 12, 13, 14], which have been repeatedly reviewed [3, 15, 16, 17]. However, from only one of these trials data on the potential effect of serum IgG trough levels on the incidence of infections are reported: Griffiths et al. (1989) [11] state that the incidence of serious bacterial infections showed a trend to be associated with an IgG level < 6.4 g/L (p = 0.046). 

Privigen (CSL Behring, King of Prussia, PA, USA) is a ready-to-use liquid preparation of 10% polyvalent human IgG for intravenous administration. It is prepared from thousands of plasma donations, resulting in a broad spectrum of antibody specificities. The IgG subclass distribution in Privigen is similar to that found in normal serum. Stabilization with L-proline at pH 4.8 diminishes the formation of idiotype/anti-idiotype dimers and preserves IgG functional activity without refrigeration [18, 19]. 

Previous clinical trials have demonstrated efficacy and tolerability of Privigen in both children and adults with primary immunodeficiency [18], immune thrombocytopenia [19], and chronic inflammatory demyelinating polyneuropathy [20]. However, there is a scarcity of data on the efficacy of Privigen in secondary immunodeficiencies (SID) and especially on the correlation between the serum IgG concentrations under treatment with Privigen and the rate of infections. The current analysis was performed in order to fill this gap. The data presented here are from a non-interventional study on the effectiveness and tolerability of Privigen substitution therapy in CLL patients with SID. 

## Materials and methods 

This non-interventional, prospective study was conducted in 31 centers in Germany and 1 center in Austria. The study has been approved by an independent ethics committee and has been registered in the non-interventional studies registry at the Paul Ehrlich Institute (German federal authority for vaccines and biomedicines; study code PVG-CLL-RTX-09; NIS-Nr. 125). The study was funded by CSL Behring Hattersheim, the German affiliate of the manufacturer of Privigen. Adult patients (≥ 18 years) diagnosed with CLL, secondary hypogammaglobulinemia, and recurrent infections, for whom their physicians had made a prior decision for treatment with Privigen irrespective of the potential study participation, were included in the study after informed consent had been obtained. Due to the non-interventional character of the study, no additional examinations other than those foreseen within local routine care and driven by medical need were carried out. Patients were planned to be observed for 12 months, thereby leveling out seasonal effects on the incidence of infections. 

For the current investigation, the association between the serum IgG concentrations during treatment and the clinical effectiveness of Privigen in terms of frequency and severity of infections was analyzed (“effectiveness analysis”). Only patients with both evaluable serum IgG trough levels (measured not earlier than 3 days before the subsequent infusion) and a minimum of 180 days in evaluable treatment cycles were included in this analysis. 

**Figure 1.**
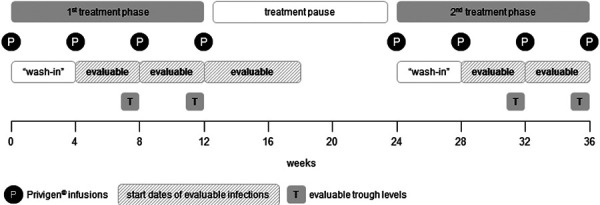
Illustration of the analysis rules based on an exemplary treatment course of 36 weeks with 4 Privigen infusions at intervals of 4 weeks (“treatment cycles”), a pause of 3 months, and then 4 further infusions.

Moreover, the following definitions and rules were introduced for the analysis (Figure 1):

- A treatment cycle was defined as the time period between two Privigen infusions (dosing interval) if this interval spanned at least 7 days. If a Privigen infusion followed less than 7 days after the previous infusion, it was considered part of the same treatment cycle.
- “Wash-in period”: The first treatment cycle after study inclusion, and the first treatment cycle after a treatment pause of more than 2 months were considered as “wash-in periods” and not included in the analysis (e.g., when calculating infection rates per 10 patient years).
- Infections that were already ongoing at baseline were not included in the analysis, irrespective if they persisted until an evaluable treatment cycle.
- Infections that began > 6 weeks (~ 2 half-lives of IgG) after the previous infusion (e.g., in a treatment pause of > 2 months or after the last Privigen infusion) were not included in the analysis.
- Only infections that required specific antimicrobial treatment (antibiotics, antifungals, or antivirals) were included in the analysis.

The evaluable patients were stratified according to their mean post-baseline serum IgG trough levels in a group with lower IgG trough levels and a group with higher IgG trough levels, using a cut-off value of 5.0 g/L, often considered to be a minimum for effective infection control in substitution treatment of SID patients [21, 22]. 

For the analysis of the tolerability of Privigen, adverse events and investigator assessments of overall tolerability in all treatment cycles of all 160 included patients were considered. 

### Statistics 

Quantitative data were analyzed by statistical parameters such as N, N missing, mean, standard deviation, 95% confidence interval, minimum, maximum, median and the 25% and 75% percentile. Qualitative and ordinal data were analyzed by absolute and relative frequency. p-values were calculated using t-test, Fisher’s exact test or negative binomial regression, as appropriate. p-values were not adjusted for multiplicity, and a significance level of 5% (2-sided) was used. The log time on treatment was used as offset in the negative binomial model; age and sex were included as covariates. All analyses were performed using SAS software, version 9.4 (SAS Institute, Cary, NC, USA). 

## Results 

### Patients 

The study was conducted between October 2010 and August 2016. 

160 patients from 31 centers in Germany and 1 in Austria were included in the non-interventional study, receiving a total of 1,465 Privigen infusions (dosage at the discretion of the treating physician). 

89 patients had both evaluable serum IgG trough levels and a minimum of 180 days in evaluable treatment periods and could therefore be included in the effectiveness analysis. On average, 5 evaluable IgG trough levels per patient were available (range 1 – 10). Characteristics of the 89 patients at baseline are summarized in Table 1. 

The two patient groups in the effectiveness analysis – one with mean IgG trough levels ≤ 5.0 g/L (n = 17) and one with mean IgG trough levels > 5.0 g/L (n = 72) – were similar in terms of gender, age, weight, time since CLL diagnosis, and Binet stage. However, in the group with higher IgG trough levels, considerably more patients had had previous treatment with immunoglobulins within the 12 months before inclusion in the study as compared with the group with low IgG trough levels (72 vs. 29%, p = 0.002). Therefore, comparisons of data from within the study period with data prior to inclusion (pre-post group comparisons) would not be conclusive and are not presented. 

### Treatment 

The 89 patients in the effectiveness analysis received 1,044 Privigen infusions (on average 11.7 infusions per patient; range 9 – 12). A total of 840 treatment cycles were evaluable with regard to effectiveness. Table 2 shows relevant treatment parameters in the group with higher IgG trough levels and in the group with lower IgG trough levels. The patients in the group with higher IgG trough levels received signiﬁcantly higher Privigen doses. 

The average monthly dose in the group with lower IgG trough levels was 0.15 g/kg body weight (BW)/month and was thus lower than the dosage recommended in the summary of product characteristics [18] and in German guidelines [23] (0.2 – 0.4 g/kg BW every 3 – 4 weeks). The range was 0.11 – 0.24 g/kg BW/month, and in only 12% of the patients, the dosage was within the recommended range. 

The average monthly dose in the group with higher IgG trough levels was 0.20 g/kg BW/month, which is just at the lower margin of the recommendations. The range was 0.08 – 0.47 g/kg BW/month. 

### Effectiveness 

**Figure 2.**
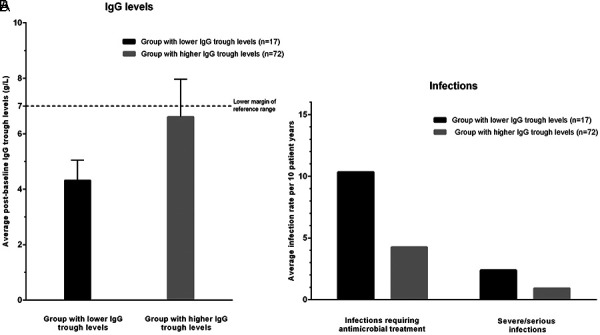
Serum IgG trough levels (A; g/L; mean + SD) and infection rates (B; mean) in the group with lower IgG trough levels (≤ 5.0 g/L) and in the group with higher IgG trough levels (> 5.0 g/L); rates were calculated using the evaluable treatment periods as illustrated in Figure 1; p-value for group comparison: 0.038 (negative binomial regression model for all infections requiring antimicrobial treatment). The lower margin of reference range for serum IgG in adults is given as 7 g/L [31, 32].

The average post-baseline serum lgG trough levels (stratiﬁcation variable with a cut-off value of 5.0 g/L) were 6.6 g/L (mean of means; range 5.01 – 11.93 g/L) in the group with higher IgG trough levels (N = 72), and 4.3 g/L (range 2.06 – 4.99 g/L) in the group with lower IgG trough levels (N = 17) (Figure 2A). Following the above-described analysis rules, a total of 840 treatment cycles in 89 patients could be analyzed with regard to effectiveness (68 patient years; mean of the median cycle durations: 29 days). During these cycles, the following 36 infections requiring speciﬁc antimicrobial treatment (antibiotics, antifungals, antivirals) were documented: upper respiratory tract infections (N = 12), acute bronchitis or acute exacerbation of chronic bronchitis (N = 3), pneumonia (N = 4), urinary tract infections (N = 11), and other infections (N = 6). Eight infections were rated as severe (by the investigator on a three-level scale with “mild”, “moderate” and “severe”) or serious (referring to an FDA Guidance [24] with a list of infections qualifying per se as serious): 5 × pneumonia (“serious”), 2 × urinary tract infection, 1 × enteritis, 1 × bronchitis. The rates both for all evaluable infections and for the severe or serious infections were more than twice as high in the group with lower IgG trough levels than in the group with higher IgG trough levels (10.3 vs. 4.4 and 2.5 vs. 1.0 infections per 10 patient years) (Figure 2B). The result is statistically signiﬁcant for all evaluable infections (p = 0.038 using negative binomial regression without covariates; p = 0.042 using negative binomial regression including age and sex as covariates). 

### Tolerability 

In the total study population of 160 patients, 1,465 infusions were documented. For 18 infusions (1.2%; 16 patients), adverse events assessed as possibly or probably related to Privigen by the treating physician (= adverse drug reactions (ADRs)) were reported. In 2 cases, the event was rated serious (0.14%). Events that occurred at least in 2 instances included chills (6 events), back pain (3), sweating (3), allergic reaction (2), dyspnea (2), facial ﬂushing (2), skin rash (2), and increased temperature (2). The overall tolerability was assessed by the treating physicians as “very good” in 69 cases (43%), good in 77 cases (48%), moderate in 5 cases (3%), rather poor in 5 cases (3%), insufﬁcient in 3 cases (2%), and not evaluable in 1 case (1%). 

As regards the 89 patients included in the effectiveness analysis, those in the group with higher IgG trough levels (receiving higher dosages, n = 72) did not have a higher rate of reported adverse reactions to Privigen than those in the group with lower IgG trough levels (receiving lower dosages, n = 17): adverse reactions to Privigen were observed in 0.4% and 0.5% of infusions in these groups (n = 846 and 198, respectively; p = 0.57). For reasons discussed below, the rate of adverse events possibly or probably related to Privigen was higher in the patient population excluded from the effectiveness analysis (n = 71; ADRs in 12/71 = 17% of the patients, and in 14/421 = 3.3% of the infusions). The baseline characteristics of the 71 patients who were excluded from the effectiveness analysis were similar to the patients who were included: 58% of the patients were male; mean age was 69.7 ± 8.2 years; mean weight was 78.0 ± 16.1 kg; Binet stage was A in 45% of the patients, B in 38%, and C in 10%. The mean total observation period was 186 ± 131 days and considerably shorter than in the patients who were included in the effectiveness analysis (Table 2); median duration of treatment cycles was 30.2 ± 10.6 days (mean of medians); mean Privigen dose per treatment cycle was 14.6 ± 7.1 g. 

**Figure 3.**
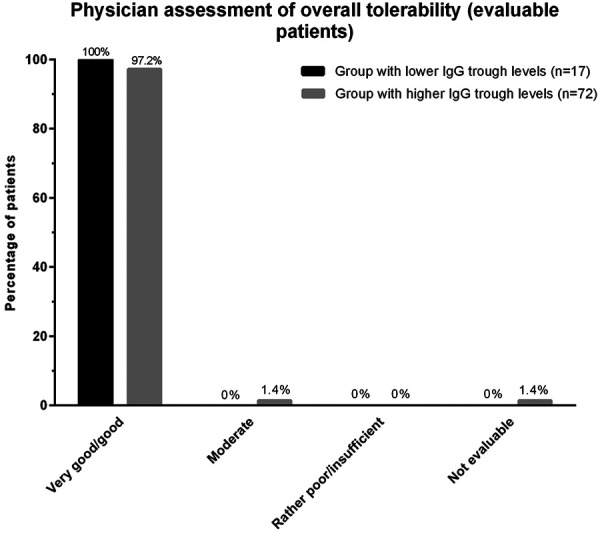
Assessment of overall tolerability of Privigen by the treating physicians in the group with lower IgG trough levels (≤ 5.0 g/L) and in the group with higher IgG trough levels (> 5.0 g/L).

The overall tolerability of Privigen was very similar in the two IgG subgroups: In 91% of the patients, the overall tolerability was assessed as “very good” or “good” by the treating physicians (Figure 3). One patient in the group with higher IgG trough levels discontinued treatment because of an adverse event with a suspected causal relationship to Privigen (pruritus), and 1 patient in the group with lower IgG trough levels discontinued treatment because of inadequate clinical response. 

There was no obligation for the investigators to report adverse events which were not suspected to be ADRs, whether they were serious or not. The only exception from this were deaths, which had to be reported irrespective of causality. Five patients died within 2 months after their last Privigen infusion. In 2 cases, the underlying malignant disease was reported as the cause of death; in 1 case, the patient died of sepsis with multiorgan failure; in 2 cases, the cause of death was not reported. In the latter cases, the tolerability of Privigen was reported as “good”, and the investigators did not relate the deaths to Privigen. 

## Discussion 

The current analysis aimed to provide insight into the association between serum IgG concentrations and the infection rates during treatment with Privigen. For this purpose, the patient cohort of 89 evaluable patients was divided into two subgroups, based on their post-baseline serum IgG trough levels. As a cut-off, 5.0 g/L was chosen, which at times has been considered to be a minimum for effective infection control [21, 22]. In the group with higher IgG trough levels (> 5.0 g/L), significantly fewer infections requiring antimicrobial treatment were observed than in the group with lower IgG trough levels. The patients with higher IgG levels had also a clear advantage regarding severe and serious infections. 

It should be noted that a serum IgG trough level of 5 g/L – although proven useful in the analysis to subdivide our study population in a group with lower IgG levels and higher infections rates on the one hand, and a group with higher IgG levels and lower infections rates on the other hand – is not necessarily a suitable target for dosing decisions. One should keep in mind that the patient group with lower infection rates had an average IgG trough level of 6.6 g/L, which is well above the chosen threshold of 5 g/L and not far from the lower margin of the reference range of 7 – 16 g/L. 

Importantly, in 2018, the EMA revised their recommendations for the target IgG trough levels in IVIG-treated patients with primary immunodeficiencies (PID) from previously “at least 5 to 6 g/L” (2013) to “at least 6 g/L or within the normal reference range for the population age” [22, 25]. This reflects a series of studies showing a significant inverse correlation between IgG trough levels and serious as well as non-serious infections in PID patients [26, 27, 28]. The most important result of the meta-analysis by Orange et al. [26] was that in the range between 5 and 10 g/L, an incremental increase of the serum IgG trough level by 1 g/L was associated with a reduction of the pneumonia incidence by 27% on the average. 

Data on such a correlation are scarce for (SID) [11], nonetheless in 2020, the EMA approved the same wording for the target serum IgG level in the treatment of SID with Privigen: “at least 6 g/l or within the normal reference range for the population age” [29]. With an average IgG trough level of 6.6 g/L in the group with higher serum IgG trough levels and lower infections rates, the current study supports this recommendation. 

Although the serum IgG levels during IVIG treatment are also influenced by other factors (underlying IgG production, IgG metabolism, possibly protein loss), the Privigen dosage was a major determinant of the IgG levels in our analysis: the mean monthly Privigen dose was nearly 50% higher in the group with higher IgG trough levels than in the group with lower IgG trough levels (15.3 vs. 10.4 g; p = 0.02). The vast majority (88%) of the patients in the group with lower IgG trough levels and higher infection rates were treated with a fixed single dose of 10 g per treatment cycle (usually 4 weeks), which in an average-size adult is lower than the recommended dosage according to the Privigen Summary of Product Characteristics (SmPC) and German guidelines (0.2 – 0.4 g/kg BW every 3 – 4 weeks) [18, 23, 30]. In fact, a patient weighing 75 kg would require 15 – 30 g IVIG every 3 – 4 weeks according to the official recommendations. In the group with lower IgG trough levels, however, only 12% of the patients had a dosage reaching the recommended minimum of 0.2 g/kg every 4 weeks, so underdosing may have played an essential role for the significantly higher infection rates in these patients. 

The rates of adverse events possibly or probably related to Privigen (= ADRs) were low in both patient groups which were investigated in the effectiveness analysis (total of 89 out of 160 patients): In the group with lower IgG trough levels (n = 17), ADRs were reported in 0.5% of the infusions, while in the group with higher IgG trough levels (n = 72), it was 0.4% (p = 0.57), so the higher doses used in the latter group were not reflected in a decreased tolerability. When interpreting these low rates, however, it has to be considered that some of the inclusion criteria applied for the effectiveness analysis (89 patients included) favored patients with good tolerability. This is quite evident for the criterion “≥ 180 days in evaluable treatment cycles”, since patients who do not tolerate the treatment well tend to discontinue earlier. In accordance with this, the rate of adverse events possibly or probably related to Privigen was markedly higher in the patient population excluded from the effectiveness analysis (n = 71; ADRs in 3.3% of all infusions), but even in this population, for 83% of the patients (59/71) no ADR was recorded. 

## Conclusion 

Our analysis revealed a clear inverse association between serum IgG trough levels and the rate of infections in IVIG-treated CLL patients with SID. Low IgG trough levels were associated with dosages below the recommendations in the Privigen SmPC and in guidelines. Keeping in mind that infections are a major cause of death in CLL, we suggest that IgG trough levels be carefully monitored during IVIG substitution treatment in order to avoid possible undertreatment since there is a signiﬁcant increase in the number of infections, including severe and serious infections, associated with low IgG levels. 

## Study registration 

The study has been registered in the non-interventional studies registry at the Paul Ehrlich Institute (German federal authority for vaccines and biomedicines; study code PVG-CLL-RTX-09; NIS-Nr. 125). 

## Authors’ contributions 

Study concept and design: Otremba, Pfründer; Recruitment of patients: Otremba, Haslbauer, Reiser, Weide; Analysis: Obermeier, Pfründer; Drafting and finalization of the manuscript: Otremba, Haslbauer, Reiser, Weide, Pfründer. 

## Acknowledgment 

Medical writing support was provided by GKM Gesellschaft für Therapieforschung mbH (Munich, Germany). 

## Funding 

The study was funded by CSL Behring (Hattersheim, Germany), the manufacturer of Privigen. 

## Conflict of interest 

BO has received consultancy honoraria from CSL Behring GmbH for his role as the primary investigator of this study; FH has received speaker’s fees from CSL Behring GmbH for presenting results at a conference; BO, FH, MR, and RW have received investigators’ fees for recruiting patients and documenting their treatment courses; MO is an employee of GKM Gesellschaft für Therapieforschung mbH, supporting CSL Behring GmbH as a CRO; DP is an employee of CSL Behring GmbH. 

**Table 1.** Patient characteristics at baseline.

	Group with lower (≤ 5.0 g/L) IgG trough levels (n = 17)	Group with higher (> 5.0 g/L) IgG trough levels (n = 72)	Unadjusted p-value (comparison of groups with lower vs. higher IgG trough levels)
Gender			0.79
Male	8 (47.1%)	39 (54.2%)	
Female	9 (52.9%)	33 (45.8%)	
Age (mean ± SD)	72.2 ± 9.5 years	68.8 ± 9.1 years	0.18
Weight (mean ± SD)	75.0 ± 13.9 kg	78.4 ± 15.7 kg	0.41
Time since CLL diagnosis (mean ± SD)	6.8 ± 4.3 years	9.0 ± 6.8 years	0.22
Binet stage			
A	7 (41.2%)	34 (47.2%)	0.79 (A vs. B and C)
B	6 (35.3%)	27 (37.5%)	
C	4 (23.5%)	11 (15.3%)	0.47 (C vs. A and B)
IgG treatment within 12 months before study entry	5 (29.4%)	52 (72.2%)	0.0016

IgG = immunoglobulin G; CLL = chronic lymphocytic leukemia.

**Table 2.** Details on Privigen treatment.

	Group with lower (≤ 5.0 g/L) IgG trough levels (n = 17)	Group with higher (> 5.0 g/L) IgG trough levels (n = 72)	Unadjusted p-value
Total observation period (mean ± SD)	321 ± 30 days	350 ± 67 days	0.084
Total duration of evaluable treatment periods (mean ± SD)	277 ± 27 days	282 ± 37 days	0.61
Median duration of a treatment cycle (mean ± SD)	28.1 ± 0.2 days	28.9 ± 5.3 days	0.40
Mean dose per cycle (mean ± SD)	10.4 ± 1.2 g	15.3 ± 8.6 g	0.020
Mean weight-based monthly dose (mean ± 95% CL)	0.15 ± 0.03 g/kg BW/month	0.20 ± 0.10 g/kg BW/month	0.036
Percentage of patients on a fixed single Privigen dose of 10.0 g per cycle*	88.2%	55.6%	0.013

*Differing loading dose possible. CL = confidence limits.
